# Supplementary material for: HIVprotI: an integrated web based platform for prediction and design of HIV proteins inhibitors
Source: J Cheminform. 2018 Mar 9;10:12. doi: 10.1186/s13321-018-0266-y (PMC5845081; doi:10.1186/s13321-018-0266-y)
Supplement: Supplementary file 2 — Additional file 2. Source code of HIVProtI web server. [file 13321_2018_266_MOESM2_ESM.zip › HIVprotI_Source-code/hivprotI/weka-3-6-6/doc/weka/classifiers/functions/supportVector/CachedKernel.html]

CachedKernel


---


|  |  |  |
| --- | --- | --- |
| **Overview** \| **Package** \| **Class** \| **Tree** \| **Deprecated** \| **Index** \| **Help** \| **Weka's home** | |  |
| PREV CLASS   **NEXT CLASS** | **FRAMES**    **NO FRAMES**     **All Classes** |  |
| SUMMARY: NESTED \| FIELD \| CONSTR \| METHOD | DETAIL: FIELD \| CONSTR \| METHOD |  |


---


## weka.classifiers.functions.supportVector Class CachedKernel

```
java.lang.Object
  weka.classifiers.functions.supportVector.Kernel
      weka.classifiers.functions.supportVector.CachedKernel
```

**All Implemented Interfaces:**: java.io.Serializable, CapabilitiesHandler, OptionHandler, RevisionHandler

**Direct Known Subclasses:**: PolyKernel, Puk, RBFKernel

---

``` public abstract class CachedKernel extends Kernel ```

Base class for RBFKernel and PolyKernel that implements a simple LRU.
(least-recently-used) cache if the cache size is set to a value > 0.
Otherwise it uses a full cache.

**Version:**
:   $Revision: 4549 $

**Author:**
:   Eibe Frank (eibe@cs.waikato.ac.nz), Shane Legg (shane@intelligenesis.net) (sparse vector code), Stuart Inglis (stuart@reeltwo.com) (sparse vector code), J. Lindgren (jtlindgr{at}cs.helsinki.fi) (RBF kernel), Steven Hugg (hugg@fasterlight.com) (refactored, LRU cache), Bernhard Pfahringer (bernhard@cs.waikato.ac.nz) (full cache)

**See Also:**: Serialized Form

---

**Constructor Summary**

`CachedKernel()`
  
          default constructor - does nothing.


| **Method Summary** | |
| --- | --- |
| `void` | `buildKernel(Instances data)`             builds the kernel with the given data. |
| `java.lang.String` | `cacheSizeTipText()`             Returns the tip text for this property |
| `void` | `clean()`             Frees the cache used by the kernel. |
| `double` | `eval(int id1, int id2, Instance inst1)`             Implements the abstract function of Kernel using the cache. |
| `int` | `getCacheSize()`             Gets the size of the cache |
| `java.lang.String[]` | `getOptions()`             Gets the current settings of the Kernel. |
| `java.util.Enumeration` | `listOptions()`             Returns an enumeration describing the available options. |
| `int` | `numCacheHits()`             Returns the number of cache hits on dot products. |
| `int` | `numEvals()`             Returns the number of time Eval has been called. |
| `void` | `setCacheSize(int value)`             Sets the size of the cache to use (a prime number) |
| `void` | `setOptions(java.lang.String[] options)`             Parses a given list of options. |

**Methods inherited from class weka.classifiers.functions.supportVector.Kernel**

`checksTurnedOffTipText, debugTipText, forName, getCapabilities, getChecksTurnedOff, getDebug, getRevision, globalInfo, makeCopies, makeCopy, setChecksTurnedOff, setDebug`

**Methods inherited from class java.lang.Object**

`equals, getClass, hashCode, notify, notifyAll, toString, wait, wait, wait`

**Constructor Detail**

### CachedKernel

```
public CachedKernel()
```

:   default constructor - does nothing.


**Method Detail**

### listOptions

```
public java.util.Enumeration listOptions()
```

:   Returns an enumeration describing the available options.

    :   **Specified by:**: `listOptions` in interface `OptionHandler` **Overrides:**: `listOptions` in class `Kernel`
    :   **Returns:**: an enumeration of all the available options.

---


### setOptions

```
public void setOptions(java.lang.String[] options)
                throws java.lang.Exception
```

:   Parses a given list of options.

    :   **Specified by:**: `setOptions` in interface `OptionHandler` **Overrides:**: `setOptions` in class `Kernel`
    :   **Parameters:**: `options` - the list of options as an array of strings **Throws:**: `java.lang.Exception` - if an option is not supported

---


### getOptions

```
public java.lang.String[] getOptions()
```

:   Gets the current settings of the Kernel.

    :   **Specified by:**: `getOptions` in interface `OptionHandler` **Overrides:**: `getOptions` in class `Kernel`
    :   **Returns:**: an array of strings suitable for passing to setOptions

---


### eval

```
public double eval(int id1,
                   int id2,
                   Instance inst1)
            throws java.lang.Exception
```

:   Implements the abstract function of Kernel using the cache. This method
    uses the evaluate() method to do the actual dot product.

    :   **Specified by:**: `eval` in class `Kernel`
    :   **Parameters:**: `id1` - the index of the first instance in the dataset: `id2` - the index of the second instance in the dataset: `inst1` - the instance corresponding to id1 (used if id1 == -1) **Returns:**: the result of the kernel function **Throws:**: `java.lang.Exception` - if something goes wrong

---


### numEvals

```
public int numEvals()
```

:   Returns the number of time Eval has been called.

    :   **Specified by:**: `numEvals` in class `Kernel`
    :   **Returns:**: the number of kernel evaluation.

---


### numCacheHits

```
public int numCacheHits()
```

:   Returns the number of cache hits on dot products.

    :   **Specified by:**: `numCacheHits` in class `Kernel`
    :   **Returns:**: the number of cache hits.

---


### clean

```
public void clean()
```

:   Frees the cache used by the kernel.

    :   **Specified by:**: `clean` in class `Kernel`

---


### setCacheSize

```
public void setCacheSize(int value)
```

:   Sets the size of the cache to use (a prime number)

    :   **Parameters:**: `value` - the size of the cache

---


### getCacheSize

```
public int getCacheSize()
```

:   Gets the size of the cache

    :   **Returns:**: the cache size

---


### cacheSizeTipText

```
public java.lang.String cacheSizeTipText()
```

:   Returns the tip text for this property

    :   **Returns:**: tip text for this property suitable for displaying in the explorer/experimenter gui

---


### buildKernel

```
public void buildKernel(Instances data)
                 throws java.lang.Exception
```

:   builds the kernel with the given data. Initializes the kernel cache.
    The actual size of the cache in bytes is (64 \* cacheSize).

    :   **Overrides:**: `buildKernel` in class `Kernel`
    :   **Parameters:**: `data` - the data to base the kernel on **Throws:**: `java.lang.Exception` - if something goes wrong


---


|  |  |  |
| --- | --- | --- |
| **Overview** \| **Package** \| **Class** \| **Tree** \| **Deprecated** \| **Index** \| **Help** \| **Weka's home** | |  |
| PREV CLASS   **NEXT CLASS** | **FRAMES**    **NO FRAMES**     **All Classes** |  |
| SUMMARY: NESTED \| FIELD \| CONSTR \| METHOD | DETAIL: FIELD \| CONSTR \| METHOD |  |


---
